# Poly[[bis­(μ_2_-4,4′-bipyridine)[μ_2_-(2,4-dichloro­phen­oxy)acetato]copper(I)] nitrate]

**DOI:** 10.1107/S1600536810002229

**Published:** 2010-01-23

**Authors:** Shi-Zhu Liu

**Affiliations:** a School of Chemistry and Environment, South China Normal University, Guangzhou 510631, People’s Republic of China

## Abstract

The title compound, {[Cu_2_(C_8_H_5_Cl_2_O_3_)(C_10_H_8_N_2_)_2_]NO_3_}_*n*_ was prepared by reacting copper(II) nitrate with 4,4′-bipyridine (4,4′-bipy) and (2,4-dichloro­phen­oxy)acetic acid under solvothermal conditions. Each of two copper(I) atoms in the asymmetric unit is three-coordinated by two N atoms from two 4,4′-bipy ligands and one O atom from the (2,4-dichloro­phen­oxy)acetate ligand. As both ligands act as bridging ligands, a double-stranded chain structure is observed.

## Related literature

For coordination polymers incorporating either 4,4′bipy or phenoxy­acetato ligands and Cu(I) or Cu(II), see: Biswas *et al.* (2007 ▶); Bourne & Moitsheki (2007 ▶); Huang *et al.* (2008 ▶); Mo *et al.* (2009 ▶); Smith *et al.* (1981 ▶). 
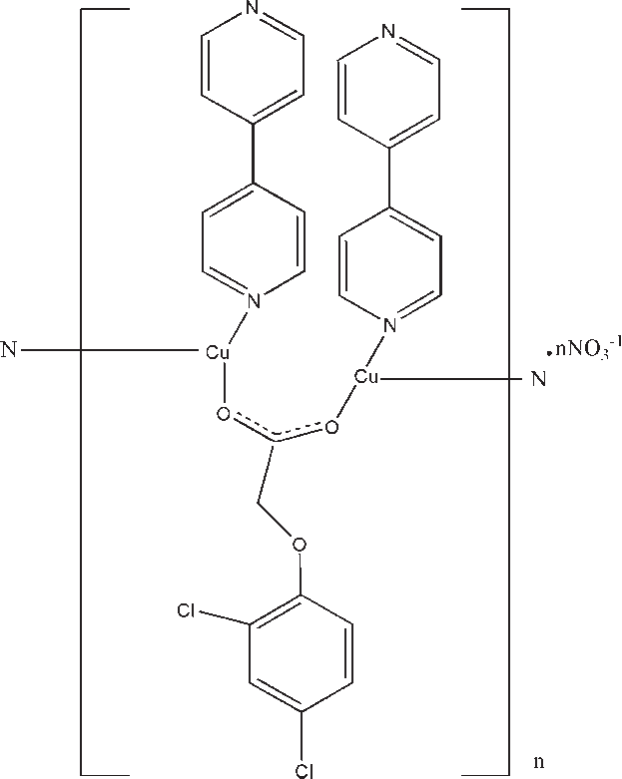


## Experimental

### 

#### Crystal data

[Cu_2_(C_8_H_5_Cl_2_O_3_)(C_10_H_8_N_2_)_2_]NO_3_
*M* *_r_* = 721.5
Monoclinic, 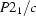

*a* = 10.598 (2) Å
*b* = 18.552 (3) Å
*c* = 15.212 (3) Å
β = 108.835 (2)°
*V* = 2830.9 (8) Å^3^
*Z* = 4
Mo *K*α radiation
μ = 1.74 mm^−1^
*T* = 296 K
0.15 × 0.13 × 0.08 mm

#### Data collection

Bruker APEXII area-detector diffractometer
Absorption correction: multi-scan (*SADABS*; Sheldrick, 1996 ▶) *T*_min_ = 0.883, *T*_max_ = 0.935
14129 measured reflections
5085 independent reflections
3787 reflections with *I* > 2σ(*I*)
*R*_int_ = 0.032

#### Refinement

*R*[*F* ^2^ > 2σ(*F* ^2^)] = 0.049
*wR*(*F* ^2^) = 0.143
*S* = 1.04
5085 reflections
388 parameters
H-atom parameters constrained
Δρ_max_ = 0.89 e Å^−3^
Δρ_min_ = −0.68 e Å^−3^

### 

Data collection: *APEX2* (Bruker, 2004 ▶); cell refinement: *SAINT* (Bruker, 2004 ▶); data reduction: *SAINT*; program(s) used to solve structure: *SHELXS97* (Sheldrick, 2008 ▶); program(s) used to refine structure: *SHELXL97* (Sheldrick, 2008 ▶); molecular graphics: *XP* in *SHELXTL* (Sheldrick, 2008 ▶); software used to prepare material for publication: *SHELXL97*.

## Supplementary Material

Crystal structure: contains datablocks I, global. DOI: 10.1107/S1600536810002229/im2172sup1.cif

Structure factors: contains datablocks I. DOI: 10.1107/S1600536810002229/im2172Isup2.hkl

Additional supplementary materials:  crystallographic information; 3D view; checkCIF report

## Figures and Tables

**Fig. 1.**
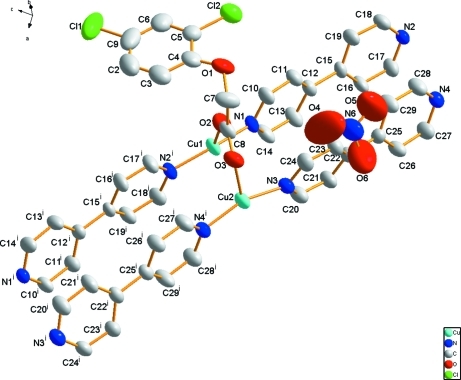
The asymmetric unit of the title compound, showing displacement ellipsoids at 50% probability for non-H atoms. Symmetry code: i 1 + x, 0.5 - y, 1/2 + z.

**Fig. 2.**
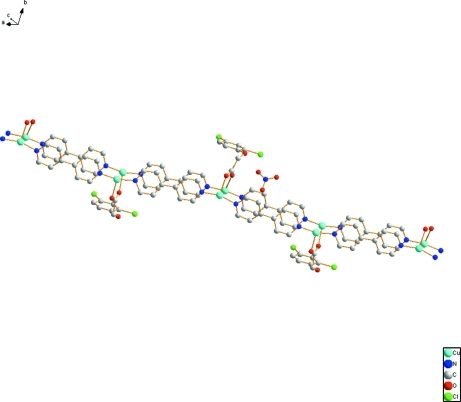
A view of the one-dimensional polymeric chain structure of the title compound. Hydrogen atoms are omitted for clarity.

## supplementary crystallographic information

### Comment

The design and synthesis of inorganic-organic composite coordination polymers exhibiting novel structures and properties is an intensively studied field of chemical research. 4,4'-bipyridine is a ligand that is particularly suited for constructing frameworks that possess hydrophobic pores and channels with potentially useful inclusion properties, including size and shape specificity (Bourne & Moitsheki, 2007; Huang *et al.*, 2008; Mo *et al.*, 2009; Biswas *et al.*, 2007). 2,4-Dichlorophenoxyacetato ligands have been described to act as bridging in ligands in a typical Cu(II) paddlewheel complex (Smith *et al.*, 1981).

The title compound (Fig. 1) was prepared from copper(II) nitrate, 4,4'-bipyridine (4,4'-bipy) and 2,4-dichlorophenoxyacetic acid under solvothermal conditions with ethanol most probably acting as the reducing agent for copper(II). A rufous colored block shaped crystal of the resulting copper(I) complex with a 2,4-dichlorophenoxyacetato ligand was characterized by single-crystal X-ray analysis. It reveals that each of two copper(I) centers is three-coordinated by two N atoms from two 4,4'-bipy ligands and one O atom from a bridging 2,4-dichlorophenoxyacetato ligand. The copper(I) coordination units are additionally connected by bridging 4,4'-bipy ligands, generating a one-dimensional polymeric chain strurcture (Fig. 2). A doubly stranded chain is observed due to the coordination of the copper(I) centers to one 2,4-dichlorophenoxyacetato ligand.

### Experimental

A mixture of Cu(NO_3_)_2_ × 5 H_2_O (0.121 g, 0.44 mmol), 4,4'-bipyridine × 2 H_2_O (0.096 g, 0.5 mmol) and 2,4-dichlorophenoxyacetic acid (0.22 g, 1 mmol) in 8 ml of an ethanol/H_2_O mixture (*v/v* 1/7) was stirred vigorously for 10 min and then sealed in a 25 ml teflon-lined stainless-steel autoclave. The autoclave was heated to 403 K for 2 days and was then slowly cooled to room temperature with a rate of 6 K/h. The product was collected by filtration, washed with water and air-dried. Rufous block shaped crystals suitable for X-ray analysis were obtained in *ca* 26.8% yield based on Cu.

### Refinement

All non-hydrogen atoms were refined with anisotropic displacement parameters. Hydrogen atoms attached to carbon were placed in geometrically idealized positions and refined using a riding modelwith *U*_iso_(H) = 1.2 *U*_eq_(C) for H atoms of the aromatic rings.

### Crystal data

**Table:**

[Cu_2_(C_8_H_5_Cl_2_O_3_)(C_10_H_8_N_2_)_2_]NO_3_	*F*(000) = 1456
*M**_r_* = 721.5	*D*_x_ = 1.693 Mg m^−^^3^
Monoclinic, *P*2_1_/*c*	Mo *K*α radiation, λ = 0.71073 Å
Hall symbol: -P 2ybc	Cell parameters from 5085 reflections
*a* = 10.598 (2) Å	θ = 1.8–25.2°
*b* = 18.552 (3) Å	µ = 1.74 mm^−^^1^
*c* = 15.212 (3) Å	*T* = 296 K
β = 108.835 (2)°	Block, rufous
*V* = 2830.9 (8) Å^3^	0.15 × 0.13 × 0.08 mm
*Z* = 4	

### Data collection

**Table:**

Bruker APEXII area-detector diffractometer	5085 independent reflections
Radiation source: fine-focus sealed tube	3787 reflections with *I* > 2σ(*I*)
graphite	*R*_int_ = 0.032
φ and ω scan	θ_max_ = 25.2°, θ_min_ = 1.8°
Absorption correction: multi-scan (*SADABS*; Sheldrick, 1996)	*h* = −12→12
*T*_min_ = 0.883, *T*_max_ = 0.935	*k* = −19→22
14129 measured reflections	*l* = −16→18

### Refinement

**Table:**

Refinement on *F*^2^	Primary atom site location: structure-invariant direct methods
Least-squares matrix: full	Secondary atom site location: difference Fourier map
*R*[*F*^2^ > 2σ(*F*^2^)] = 0.049	Hydrogen site location: inferred from neighbouring sites
*wR*(*F*^2^) = 0.143	H-atom parameters constrained
*S* = 1.04	*w* = 1/[σ^2^(*F*_o_^2^) + (0.068*P*)^2^ + 4.0523*P*] where *P* = (*F*_o_^2^ + 2*F*_c_^2^)/3
5085 reflections	(Δ/σ)_max_ = 0.001
388 parameters	Δρ_max_ = 0.89 e Å^−^^3^
0 restraints	Δρ_min_ = −0.68 e Å^−^^3^

### Special details

**Table:**

Geometry. All e.s.d.'s (except the e.s.d. in the dihedral angle between two l.s. planes) are estimated using the full covariance matrix. The cell e.s.d.'s are taken into account individually in the estimation of e.s.d.'s in distances, angles and torsion angles; correlations between e.s.d.'s in cell parameters are only used when they are defined by crystal symmetry. An approximate (isotropic) treatment of cell e.s.d.'s is used for estimating e.s.d.'s involving l.s. planes.
Refinement. Refinement of *F*^2^ against ALL reflections. The weighted *R*-factor *wR* and goodness of fit *S* are based on *F*^2^, conventional *R*-factors *R* are based on *F*, with *F* set to zero for negative *F*^2^. The threshold expression of *F*^2^ > σ(*F*^2^) is used only for calculating *R*-factors(gt) *etc*. and is not relevant to the choice of reflections for refinement. *R*-factors based on *F*^2^ are statistically about twice as large as those based on *F*, and *R*- factors based on ALL data will be even larger.

### Fractional atomic coordinates and isotropic or equivalent isotropic displacement parameters (Å^2^)

**Table:**

	*x*	*y*	*z*	*U*_iso_*/*U*_eq_	
Cu1	0.29363 (5)	0.27588 (3)	1.15749 (4)	0.04390 (19)	
Cu2	0.48636 (5)	0.28270 (3)	1.03403 (4)	0.04604 (19)	
N3	0.3225 (3)	0.2599 (2)	0.9378 (3)	0.0444 (9)	
C22	0.0663 (4)	0.2425 (2)	0.8069 (3)	0.0394 (9)	
C13	−0.0770 (4)	0.1880 (2)	1.0032 (3)	0.0499 (12)	
H13	−0.1148	0.1422	0.9928	0.060*	
C5	0.1398 (5)	0.5119 (2)	1.2023 (4)	0.0498 (11)	
C12	−0.1511 (4)	0.2477 (2)	0.9594 (3)	0.0380 (9)	
C24	0.2591 (4)	0.3141 (3)	0.8824 (3)	0.0466 (11)	
H24	0.3019	0.3584	0.8882	0.056*	
C10	0.0413 (4)	0.3181 (2)	1.0374 (3)	0.0451 (11)	
H10	0.0815	0.3632	1.0486	0.054*	
C11	−0.0874 (4)	0.3135 (2)	0.9796 (3)	0.0441 (10)	
H11	−0.1327	0.3553	0.9535	0.053*	
C14	0.0513 (4)	0.1966 (2)	1.0613 (3)	0.0495 (12)	
H14	0.0979	0.1559	1.0900	0.059*	
C20	0.2591 (5)	0.1964 (3)	0.9247 (4)	0.0559 (13)	
H20	0.3024	0.1571	0.9596	0.067*	
C21	0.1331 (4)	0.1861 (3)	0.8623 (3)	0.0513 (12)	
H21	0.0928	0.1411	0.8575	0.062*	
C4	0.2570 (5)	0.5161 (2)	1.1810 (4)	0.0558 (13)	
C3	0.3775 (6)	0.5171 (3)	1.2524 (5)	0.0752 (17)	
H3	0.4569	0.5201	1.2390	0.090*	
C7	0.3548 (5)	0.5004 (3)	1.0594 (4)	0.0619 (14)	
H7A	0.4340	0.5263	1.0956	0.074*	
H7B	0.3364	0.5135	0.9947	0.074*	
C9	0.2614 (9)	0.5098 (3)	1.3627 (4)	0.086 (2)	
C6	0.1390 (6)	0.5089 (3)	1.2928 (4)	0.0682 (15)	
H6	0.0596	0.5063	1.3063	0.082*	
C2	0.3796 (8)	0.5138 (3)	1.3432 (5)	0.091 (2)	
H2	0.4604	0.5143	1.3913	0.110*	
N1	0.1127 (3)	0.26020 (19)	1.0789 (2)	0.0387 (8)	
C8	0.3819 (4)	0.4199 (2)	1.0702 (3)	0.0429 (10)	
O2	0.3150 (3)	0.38364 (16)	1.1069 (2)	0.0532 (8)	
O1	0.2440 (3)	0.52203 (18)	1.0888 (3)	0.0606 (9)	
Cl2	−0.01198 (13)	0.51063 (8)	1.11418 (11)	0.0687 (4)	
Cl1	0.2599 (3)	0.50305 (12)	1.47790 (13)	0.1398 (10)	
O3	0.4707 (3)	0.39830 (17)	1.0396 (2)	0.0554 (9)	
N6	0.2506 (5)	0.4773 (4)	0.7132 (5)	0.0851 (16)	
C23	0.1347 (4)	0.3077 (2)	0.8176 (3)	0.0455 (10)	
H23	0.0960	0.3470	0.7808	0.055*	
C25	−0.0720 (4)	0.2359 (2)	0.7422 (3)	0.0376 (9)	
C15	−0.2888 (4)	0.2414 (2)	0.8933 (3)	0.0357 (9)	
C26	−0.1299 (4)	0.1708 (2)	0.7098 (3)	0.0484 (11)	
H26	−0.0817	0.1284	0.7282	0.058*	
C19	−0.3701 (4)	0.3011 (2)	0.8646 (3)	0.0473 (11)	
H19	−0.3415	0.3458	0.8915	0.057*	
C16	−0.3419 (4)	0.1766 (2)	0.8556 (3)	0.0469 (11)	
H16	−0.2936	0.1343	0.8746	0.056*	
C29	−0.1522 (4)	0.2960 (2)	0.7132 (4)	0.0563 (13)	
H29	−0.1193	0.3414	0.7348	0.068*	
C18	−0.4926 (4)	0.2953 (2)	0.7970 (3)	0.0475 (11)	
H18	−0.5433	0.3368	0.7779	0.057*	
C17	−0.4666 (4)	0.1739 (2)	0.7894 (3)	0.0505 (12)	
H17	−0.5002	0.1292	0.7657	0.061*	
C27	−0.2593 (4)	0.1678 (2)	0.6498 (3)	0.0512 (12)	
H27	−0.2959	0.1229	0.6292	0.061*	
C28	−0.2795 (5)	0.2893 (2)	0.6530 (4)	0.0547 (13)	
H28	−0.3299	0.3309	0.6343	0.066*	
N2	−0.5420 (3)	0.23224 (18)	0.7575 (2)	0.0386 (8)	
N4	−0.3348 (3)	0.22657 (18)	0.6199 (3)	0.0413 (8)	
O6	0.2775 (8)	0.4203 (3)	0.6898 (5)	0.161 (3)	
O5	0.1538 (8)	0.5055 (6)	0.6659 (6)	0.229 (5)	
O4	0.3168 (8)	0.5019 (6)	0.7763 (9)	0.282 (7)	

### Atomic displacement parameters (Å^2^)

**Table:**

	*U*^11^	*U*^22^	*U*^33^	*U*^12^	*U*^13^	*U*^23^
Cu1	0.0233 (3)	0.0560 (4)	0.0418 (3)	−0.0006 (2)	−0.0041 (2)	0.0028 (2)
Cu2	0.0269 (3)	0.0546 (4)	0.0474 (4)	0.0019 (2)	−0.0008 (2)	−0.0023 (3)
N3	0.0290 (18)	0.051 (2)	0.048 (2)	0.0013 (16)	0.0043 (16)	−0.0036 (18)
C22	0.028 (2)	0.047 (2)	0.039 (2)	−0.0007 (18)	0.0044 (18)	−0.0066 (19)
C13	0.037 (2)	0.040 (2)	0.059 (3)	−0.0082 (19)	−0.003 (2)	0.013 (2)
C5	0.056 (3)	0.036 (2)	0.057 (3)	0.007 (2)	0.017 (2)	0.003 (2)
C12	0.026 (2)	0.046 (2)	0.037 (2)	−0.0006 (17)	0.0030 (17)	0.0049 (19)
C24	0.032 (2)	0.052 (3)	0.050 (3)	−0.0088 (19)	0.005 (2)	−0.002 (2)
C10	0.028 (2)	0.045 (2)	0.052 (3)	−0.0045 (18)	−0.0022 (19)	0.000 (2)
C11	0.032 (2)	0.042 (2)	0.049 (3)	0.0026 (18)	0.0001 (19)	0.005 (2)
C14	0.032 (2)	0.046 (3)	0.058 (3)	0.0006 (19)	−0.003 (2)	0.017 (2)
C20	0.040 (2)	0.045 (3)	0.067 (3)	0.004 (2)	−0.004 (2)	−0.004 (2)
C21	0.035 (2)	0.046 (3)	0.058 (3)	0.0014 (19)	−0.005 (2)	−0.004 (2)
C4	0.056 (3)	0.036 (2)	0.069 (4)	0.011 (2)	0.012 (3)	0.007 (2)
C3	0.068 (4)	0.049 (3)	0.093 (5)	0.008 (3)	0.004 (3)	−0.003 (3)
C7	0.051 (3)	0.049 (3)	0.095 (4)	0.011 (2)	0.035 (3)	0.021 (3)
C9	0.142 (7)	0.045 (3)	0.061 (4)	0.021 (4)	0.022 (4)	−0.003 (3)
C6	0.091 (4)	0.041 (3)	0.072 (4)	0.014 (3)	0.026 (3)	0.004 (3)
C2	0.095 (5)	0.063 (4)	0.084 (5)	0.016 (4)	−0.015 (4)	−0.015 (3)
N1	0.0260 (17)	0.048 (2)	0.0352 (19)	−0.0029 (15)	0.0003 (14)	0.0045 (16)
C8	0.026 (2)	0.042 (2)	0.051 (3)	−0.0041 (18)	−0.0012 (19)	0.007 (2)
O2	0.0466 (18)	0.0451 (18)	0.060 (2)	−0.0064 (14)	0.0066 (16)	0.0104 (15)
O1	0.0492 (19)	0.058 (2)	0.079 (3)	0.0196 (16)	0.0269 (18)	0.0207 (18)
Cl2	0.0513 (7)	0.0733 (9)	0.0809 (10)	0.0034 (6)	0.0205 (7)	0.0069 (7)
Cl1	0.233 (3)	0.1117 (16)	0.0609 (11)	0.0612 (17)	0.0279 (14)	−0.0073 (10)
O3	0.0343 (16)	0.0523 (19)	0.074 (2)	0.0030 (14)	0.0096 (16)	0.0018 (16)
N6	0.046 (3)	0.090 (4)	0.106 (5)	−0.011 (3)	0.006 (3)	−0.018 (4)
C23	0.034 (2)	0.049 (3)	0.046 (3)	−0.0027 (19)	0.0027 (19)	0.003 (2)
C25	0.030 (2)	0.044 (2)	0.037 (2)	−0.0019 (17)	0.0077 (18)	−0.0073 (18)
C15	0.0248 (19)	0.046 (2)	0.030 (2)	−0.0039 (17)	0.0005 (16)	0.0009 (18)
C26	0.033 (2)	0.039 (2)	0.063 (3)	0.0032 (18)	0.000 (2)	−0.003 (2)
C19	0.030 (2)	0.043 (2)	0.056 (3)	−0.0005 (18)	−0.003 (2)	−0.006 (2)
C16	0.035 (2)	0.040 (2)	0.049 (3)	0.0053 (18)	−0.010 (2)	0.001 (2)
C29	0.039 (2)	0.037 (2)	0.076 (4)	−0.0040 (19)	−0.006 (2)	−0.005 (2)
C18	0.031 (2)	0.044 (3)	0.057 (3)	0.0054 (18)	0.000 (2)	0.000 (2)
C17	0.042 (2)	0.043 (3)	0.050 (3)	−0.0029 (19)	−0.007 (2)	−0.005 (2)
C27	0.036 (2)	0.045 (3)	0.062 (3)	−0.005 (2)	0.001 (2)	−0.008 (2)
C28	0.041 (3)	0.042 (3)	0.068 (3)	0.003 (2)	−0.001 (2)	0.001 (2)
N2	0.0247 (16)	0.046 (2)	0.0380 (19)	0.0022 (14)	0.0003 (15)	−0.0057 (16)
N4	0.0308 (18)	0.045 (2)	0.042 (2)	−0.0016 (15)	0.0043 (16)	−0.0007 (16)
O6	0.190 (7)	0.083 (4)	0.160 (6)	−0.010 (4)	−0.012 (5)	−0.011 (4)
O5	0.125 (6)	0.424 (16)	0.139 (7)	0.105 (8)	0.045 (5)	−0.002 (8)
O4	0.106 (6)	0.324 (14)	0.378 (15)	−0.042 (7)	0.023 (7)	−0.232 (12)

### Geometric parameters (Å, °)

**Table:**

Cu1—N2^i^	1.913 (3)	C7—H7A	0.9700
Cu1—N1	1.926 (3)	C7—H7B	0.9700
Cu1—O2	2.180 (3)	C9—C2	1.379 (10)
Cu2—N3	1.922 (4)	C9—C6	1.387 (9)
Cu2—N4^i^	1.931 (3)	C9—Cl1	1.762 (7)
Cu2—O3	2.155 (3)	C6—H6	0.9300
N3—C20	1.339 (6)	C2—H2	0.9300
N3—C24	1.344 (6)	C8—O2	1.235 (5)
C22—C21	1.387 (6)	C8—O3	1.243 (5)
C22—C23	1.393 (6)	N6—O4	1.088 (9)
C22—C25	1.483 (5)	N6—O5	1.169 (8)
C13—C14	1.372 (6)	N6—O6	1.181 (8)
C13—C12	1.397 (6)	C23—H23	0.9300
C13—H13	0.9300	C25—C26	1.374 (6)
C5—C6	1.380 (7)	C25—C29	1.385 (6)
C5—C4	1.383 (7)	C15—C16	1.373 (6)
C5—Cl2	1.730 (5)	C15—C19	1.384 (6)
C12—C11	1.381 (6)	C26—C27	1.381 (6)
C12—C15	1.486 (5)	C26—H26	0.9300
C24—C23	1.373 (6)	C19—C18	1.376 (6)
C24—H24	0.9300	C19—H19	0.9300
C10—N1	1.348 (5)	C16—C17	1.379 (6)
C10—C11	1.366 (5)	C16—H16	0.9300
C10—H10	0.9300	C29—C28	1.370 (6)
C11—H11	0.9300	C29—H29	0.9300
C14—N1	1.332 (6)	C18—N2	1.341 (5)
C14—H14	0.9300	C18—H18	0.9300
C20—C21	1.380 (6)	C17—N2	1.339 (5)
C20—H20	0.9300	C17—H17	0.9300
C21—H21	0.9300	C27—N4	1.342 (6)
C4—O1	1.368 (6)	C27—H27	0.9300
C4—C3	1.384 (8)	C28—N4	1.326 (6)
C3—C2	1.376 (10)	C28—H28	0.9300
C3—H3	0.9300	N2—Cu1^ii^	1.913 (3)
C7—O1	1.443 (6)	N4—Cu2^ii^	1.931 (3)
C7—C8	1.519 (6)		
			
N2^i^—Cu1—N1	161.82 (15)	C9—C6—H6	121.3
N2^i^—Cu1—O2	100.44 (13)	C3—C2—C9	119.8 (6)
N1—Cu1—O2	96.62 (13)	C3—C2—H2	120.1
N3—Cu2—N4^i^	160.65 (15)	C9—C2—H2	120.1
N3—Cu2—O3	100.74 (14)	C14—N1—C10	116.6 (3)
N4^i^—Cu2—O3	97.66 (13)	C14—N1—Cu1	125.6 (3)
C20—N3—C24	116.1 (4)	C10—N1—Cu1	117.8 (3)
C20—N3—Cu2	126.3 (3)	O2—C8—O3	127.5 (4)
C24—N3—Cu2	117.4 (3)	O2—C8—C7	118.0 (4)
C21—C22—C23	116.1 (4)	O3—C8—C7	114.6 (4)
C21—C22—C25	122.9 (4)	C8—O2—Cu1	143.1 (3)
C23—C22—C25	120.9 (4)	C4—O1—C7	118.3 (4)
C14—C13—C12	120.2 (4)	C8—O3—Cu2	114.4 (3)
C14—C13—H13	119.9	O4—N6—O5	123.0 (10)
C12—C13—H13	119.9	O4—N6—O6	119.4 (8)
C6—C5—C4	122.1 (5)	O5—N6—O6	117.6 (8)
C6—C5—Cl2	117.9 (4)	C24—C23—C22	120.1 (4)
C4—C5—Cl2	120.0 (4)	C24—C23—H23	119.9
C11—C12—C13	115.9 (4)	C22—C23—H23	119.9
C11—C12—C15	121.5 (4)	C26—C25—C29	115.9 (4)
C13—C12—C15	122.6 (4)	C26—C25—C22	122.8 (4)
N3—C24—C23	123.8 (4)	C29—C25—C22	121.2 (4)
N3—C24—H24	118.1	C16—C15—C19	116.1 (4)
C23—C24—H24	118.1	C16—C15—C12	122.2 (4)
N1—C10—C11	123.0 (4)	C19—C15—C12	121.7 (4)
N1—C10—H10	118.5	C25—C26—C27	120.4 (4)
C11—C10—H10	118.5	C25—C26—H26	119.8
C10—C11—C12	120.8 (4)	C27—C26—H26	119.8
C10—C11—H11	119.6	C18—C19—C15	120.9 (4)
C12—C11—H11	119.6	C18—C19—H19	119.6
N1—C14—C13	123.4 (4)	C15—C19—H19	119.6
N1—C14—H14	118.3	C15—C16—C17	120.2 (4)
C13—C14—H14	118.3	C15—C16—H16	119.9
N3—C20—C21	123.5 (4)	C17—C16—H16	119.9
N3—C20—H20	118.2	C28—C29—C25	120.6 (4)
C21—C20—H20	118.2	C28—C29—H29	119.7
C20—C21—C22	120.3 (4)	C25—C29—H29	119.7
C20—C21—H21	119.8	N2—C18—C19	122.8 (4)
C22—C21—H21	119.8	N2—C18—H18	118.6
O1—C4—C5	116.4 (4)	C19—C18—H18	118.6
O1—C4—C3	124.4 (5)	N2—C17—C16	123.7 (4)
C5—C4—C3	119.2 (6)	N2—C17—H17	118.1
C2—C3—C4	119.9 (7)	C16—C17—H17	118.1
C2—C3—H3	120.0	N4—C27—C26	123.2 (4)
C4—C3—H3	120.0	N4—C27—H27	118.4
O1—C7—C8	112.6 (4)	C26—C27—H27	118.4
O1—C7—H7A	109.1	N4—C28—C29	123.6 (4)
C8—C7—H7A	109.1	N4—C28—H28	118.2
O1—C7—H7B	109.1	C29—C28—H28	118.2
C8—C7—H7B	109.1	C17—N2—C18	116.1 (4)
H7A—C7—H7B	107.8	C17—N2—Cu1^ii^	120.6 (3)
C2—C9—C6	121.7 (6)	C18—N2—Cu1^ii^	123.3 (3)
C2—C9—Cl1	121.2 (6)	C28—N4—C27	116.2 (4)
C6—C9—Cl1	117.1 (6)	C28—N4—Cu2^ii^	123.5 (3)
C5—C6—C9	117.3 (6)	C27—N4—Cu2^ii^	120.3 (3)
C5—C6—H6	121.3		

Symmetry codes: (i) *x*+1, −*y*+1/2, *z*+1/2; (ii) *x*−1, −*y*+1/2, *z*−1/2.
